# Facilitating and motivating factors for reporting reprehensible conduct in care: A study among nurse practitioners and physician assistants in the Netherlands

**DOI:** 10.1111/jep.13462

**Published:** 2020-08-20

**Authors:** Luppo Kuilman, Gerard Jansen, Laetitia B. Mulder, Petrie Roodbol

**Affiliations:** ^1^ Nursing Research Section, Department of Health Sciences University of Groningen, University Medical Center Groningen Groningen The Netherlands; ^2^ Department of Physician Assistant Studies Northern Arizona University, College of Health and Human Services Phoenix BMC Arizona USA; ^3^ Department of Human Resource Management & Organizational Behaviour, Faculty of Economics and Business University of Groningen Groningen The Netherlands

**Keywords:** ethics advocacy, nurse practitioner, perceived behavioural control, physician assistant, self‐efficacy, theory of planned behaviour, whistleblowing

## Abstract

**Rationale, aims and objectives:**

The aims of this study are as follows: (a) to establish whether a relationship exists between the importance that healthcare professionals attach to ethics in care and their likelihood to report reprehensible conduct committed by colleagues, and (b) to assess whether this relationship is moderated by behavioural control targeted at preventing harm.

**Method:**

In this cross‐sectional study, which was based on a convenience sample (n = 155) of nurse practitioners (NPs) and physician assistants (PAs) in the Netherlands, we measured ethics advocacy (EA) as a motivating factor (reflecting the importance that healthcare professionals attach to ethics and care) and “behavioral control targeted at preventing harm” (BCPH) as a facilitating factor. “Reporting reprehensible conduct” (RRC) was measured as a context‐specific indicator of whistleblowing intentions, consisting of two vignettes describing morally questionable behaviour committed by colleagues.

**Results:**

The propensity to report reprehensible conduct was a function of the interaction between EA and BCPH. The only group for which EA predicted RRC consisted of individuals with above‐average levels of perceived BCPH.

**Conclusion:**

The results suggest that the importance that healthcare professionals attach to ethical aspects in care is not sufficient to ensure that they will report reprehensible conduct. Such importance does not induce reporting behaviour unless the professionals also perceive themselves as having a high level of BCPH. We suggest that these insights could be helpful in training healthcare providers to cope with ethical dilemmas that they are likely to encounter in their work.

## 1 INTRODUCTION

In recent decades, healthcare professionals have increasingly been encountering moral dilemmas in their daily work. This development seems to be associated with changes in patient behaviour, as well as with factors related to stress. The role of the patient has transformed into that of a partner within the framework of shared decision‐making.^1^ As patients become more involved in the decision‐making process, conflicts are more likely to arise between their ideas and the professional opinions, norms, or values of healthcare providers. Moreover, continuous changes in the healthcare environment have generated stress factors that are more commonly experienced by all healthcare professionals, regardless of their specialization.^2^ These stress factors include: (a) staffing problems, (b) the effects of increasing efficiency demands, (c) disturbances due to increasing hierarchical power, and (d) decreased control over one's own professional conduct.^3^, ^4^

In a moral dilemma, the aforementioned factors can make it difficult to choose the right course of ethical conduct. For example, upon witnessing a moral offence, “the right thing” is to report it. In addition to a high capacity for moral reasoning,^5^ individuals need resources in order to utilize this capacity. The availability of such resources can be problematic under conditions of high work stress. In addition, it is more difficult to reach substantiated moral judgements in contexts involving conflicting interests between professionals and patients.^6^ The influence of the aforementioned stressors on the ethical decision‐making process is known to cause “moral distress”: a psychological disequilibrium occurring when the proper course of action is known, but circumstances prevent taking such action.^7^ The increasing transformation of healthcare delivery into a moral enterprise is making it more likely that the numerous dilemmas arising in the daily work of healthcare providers will complicate the process of making ethical decisions, ultimately evoking a succession of moments of moral distress. It has been described that moral distress can have deleterious outcomes, with both intrapersonal and interpersonal consequences, while also affecting the working environment. Moral distress can inflict feelings of powerlessness regarding decision‐making processes concerning treatment, thereby leading to “indecisive behaviour.”^3^ Such indecision could also occur with regard to reporting reprehensible conduct of others.

In this study, we focus on “reporting reprehensible conduct in care” as an outcome variable, exploring factors that might determine whether contemporary healthcare professionals will or will not report instances of reprehensible conduct that they might witness. We predict that the likelihood of healthcare professionals to report reprehensible conduct is determined by a combination of the extent to which they attach importance to ethics in care and their level of perceived behavioural control. We elaborate on this in the following sections.

## 2 BACKGROUND

### 2.1 Reporting Reprehensible Conduct in Care

From the perspective of compliance with the principles of ethical care, it is essential for all healthcare providers to adhere to the professional responsibility of identifying and reporting reprehensible conduct, as derived from the ethical imperative of refraining from maleficent conduct. In this study, therefore, we regard “reporting reprehensible conduct in care” (RRC) as a type of whistleblowing that is specific to the healthcare context and that involves reporting the behaviour of colleagues who violate the rules or exhibit morally questionable conduct. We define RRC as a concept that is reserved exclusively to the healthcare domain and as a planned behaviour that is specifically applicable to the individual, autonomous healthcare provider. In our definition, RRC can include either internal or external reporting. Internal reporting focuses largely on disclosing the misconduct of colleagues or superiors to the managerial layers holding ultimate responsibility within the organization. In contrast, external reporting is aimed at disclosing such misconduct to authorities outside the organization (eg, the health inspectorate or even the press).^8^ Given our view of RRC as a healthcare‐specific concept that is strongly related to the concept of whistleblowing, we also suppose that RRC may be associated with comparable consequences for healthcare professionals. More specifically, reporting reprehensible conduct can pose a serious ethical dilemma for a healthcare professional, given that such reporting is known to have consequences at both the personal level (eg, emotional, physical health, character assassination) and the professional level (eg, occupational, financial, legal).^9^

### 2.2 Factors enhancing the likelihood of reporting reprehensible conduct

Our primary hypothesis is that two antecedent factors are particularly likely to enhance the propensity to report reprehensible conduct of colleagues. The first factor is largely motivational: the importance that healthcare professionals attach to ethicality. We refer to it as “ethics advocacy (EA).” The second factor is largely related to ability: “behavioral control targeted at preventing harm (BCPH).” We predict that BCPH functions as a condition that must be fulfilled in order for the EA to have any effect. The two factors are clarified below.

#### 2.2.1 Ethics advocacy (EA)

Ethics advocacy (EA) refers to the importance that individuals attach to ethicality within the specific context of healthcare delivery. More specifically, EA entails the extent to which healthcare professionals consider it important for attention to be paid to the ethical aspects of care within their organization and during patient contact. In our operationalization, EA appears to be closely congruent to the concept of “moral identity,” which has been defined as the degree to which being a moral individual is central to one's own self‐concept. This can vary from person to person.^10^ Moral identity has been shown to predict moral cognitions, and moral action has been shown to be negatively related to the intention to engage in ethical wrongdoing^11^ and positively related to the intention to engage in whistleblowing.^12^, ^13^

Like moral identity, EA might have a positive influence on moral behaviour. More specifically, individuals with a high level of EA attach importance to the ethical aspects of care and are likely to be more motivated to devote attention to ethical aspects themselves. They are more likely to recognize situations as moral dilemmas, and they are more inclined to make morally appropriate choices. We therefore expect individuals with a strong orientation to ethics advocacy to be more targeted at preventing harm and to be more driven by the intrinsic motivation of their own moral standard of applying ethics, thus making them more likely to report reprehensible conduct. In other words, people with high EA will be more bothered by observing immoral practices and more likely to feel an urge to denounce reprehensible conduct.

It is important to note that the motivation to act morally does not necessarily lead to morally justifiable decisions. Although an individual may have a high propensity for ethics advocacy and, consequently, a strong desire to report reprehensible conduct, a certain degree of *behavioural control* is needed.

#### 2.2.2 Perceived behavioural control targeted at preventing harm

An individual who is motivated to report reprehensible conduct cannot convert this motivation into action without feeling able to do so. Individuals thus need to perceive that they have behavioural control. According to Bandura, the ways in which people behave are generally better predicted by their perceived behavioural control (or “self‐efficacy”) than by their factual skills. This is because perceived behavioural control helps individuals to determine what to do with the knowledge and skills that they have.^14^ With regard to reporting behaviour, it has been shown that self‐efficacy is positively related to the intention to report fraud that has been detected,^15^ and that perceived behavioural control is a positive predictor of whistleblowing intentions.^16^ In the current paper, we argue that perceived behavioural control has a direct effect on reporting behaviour, in addition to moderating the relationship between EA and reporting behaviour. More specifically, we reason that EA increases the likelihood of reporting reprehensible conduct, but only among people who sense that it would be easy to perform such behaviour.^17^ We therefore hypothesize that EA will more strongly increase the likelihood of reporting reprehensible conduct when perceived behavioural control is high, rather than low.

To test this hypothesis, we operationalized a construct of perceived behavioural control that is specific to the context of healthcare and in line with the most fundamental precepts of the Hippocratic oath of “First, do no harm.” As such, we introduce the measure “Behavioral control targeted at preventing harm” (BCPH).

**FIGURE 1:**
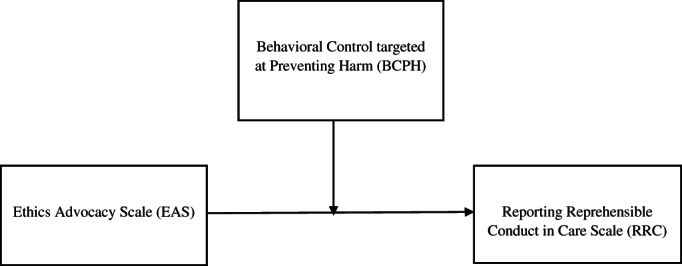
Conceptual model for simple moderation analysis. X = EAS; Y = RRC; M = BCPH. *R* ^*2*^ = .081, *F*(3, 151) = 4,49, *P* = .0047

In summary (see also Figure 1), our research has two aims: 1) to establish whether a relationship exists between attitudes toward ethics advocacy (EA, variable *X*) and the likelihood of reporting reprehensible conduct committed by colleagues (RRC, variable *Y*), and 2) to assess whether behavioural control targeted at preventing harm (BCPH, variable *M*) interacts with the relationship between *X* and *Y*.

## 3 METHOD

### 3.1 Study design, participants, and data collection

In this cross‐sectional study, we selected five PA degree programs and one NP degree program as sources for approaching alumni. In accordance with the European General Data Protection Regulation, the researchers were not granted permission to use the databases of the programs in order to retrieve the email addresses of alumni. For this reason, administrators of the programs sent the information letter concerning the study to 470 NP alumni and 426 PA alumni. By activating a hyperlink to a private web‐based system included in this letter, individual alumni were free to reveal their contact details to the researchers. When respondents granted permission to use their email addresses, this was regarded as informed consent. In all, 294 subjects (176 PAs and 118 NPs) expressed willingness to participate. Each of these subjects was sent the access key to the web‐based set of questionnaires. At the end of the online survey period (January‐March 2015), 155 respondents had completed all of the questionnaires, indicating a response rate of 52.7% (ie, 155/294). We were unable to test for selection bias, as no information was available about the alumni who did not participate. Because all of the questions in the Qualtrics online survey environment were forced choice, there were no missing data.

The dataset used in the current study was the same as the one in previous studies by Kuilman and colleagues.^18^, ^19^ Different variables were used from that pool, however, the current study focused on different research questions. In one previous study (Kuilman et al.), the “Ethics Advocacy Scale” (EAS) and the scale for “Behavioral Control targeted at Preventing Harm” (BCPH) were used for the purpose of convergent and discriminant validation.

### 3.2 Measurements

#### 3.2.1 Sociodemographic characteristics

The following background characteristics were collected for purposes of conducting tests for the comparability of the NP and PA samples: gender, age, religious beliefs, and political affiliation. Respondents were also asked to characterize their working environments as (a) “hospital,” (b) “general practice,” (c) “mental healthcare,” (d) “care for people with mental disabilities” and (e) “other.”

#### 3.2.2 Reporting reprehensible conduct in care (RRC)

Reporting behaviour was measured by presenting respondents with two vignettes (See Supporting Information, Data S1). In each of the described situations, a colleague exhibited morally questionable behaviour. After reading the vignettes, the respondents were asked to indicate the probability that they would report this behaviour, based on a 10‐cm visual analogue scale (VAS) with a minimum value of 0 and maximum of 100 at interval level.

Higher scores on the visual analogue scale indicated greater likelihood of reporting reprehensible conduct. Factor analysis revealed that the two scales were highly correlated with the underlying construct, with factor loadings of 0.80 and 0.81, respectively, explaining 69.4% of the variance. Communalities were > .6, thus suggesting that the sample size (N = 155) was good. This was corroborated by the Kaiser‐Meyer‐Olkin measure of sample adequacy (.70), which was also in the range of “good.”^20^ In the current study, the scale items were operationalized for unidimensionality rather than for internal consistency. For this reason, the degree of intercorrelation between items was used as a straightforward indicator of reliability. Unlike Cronbach's alpha, the mean inter‐item correlation (MIIC) is not dependent on the number of items in the scale. According to the guidelines of Briggs and Cheek, the optimal range for the MIIC is between 0.20 and 0.50, but it should not be less than 0.15.^21^ It therefore seems reasonable to take the upper value of the range (ie, MIIC≥.25 to ≤.55). The MIIC value of 0.34 confirmed the homogeneity of the RRC scale.

Within the regression‐based moderation model, “reporting reprehensible conduct” was estimated according to two indicators—(a) changing the waiting list for heart transplantation (Vignette 1), and (b) suspected administration of morphine (Vignette 2)—as a linear combination of the subjects' scores on both subscales.^22^, ^23^ Residual correlations between the two indicators of planned behaviour and the likelihood of reporting reprehensible conduct were allowed, as they belonged to the same measure and were assessed simultaneously.

#### 3.2.3 Ethics Advocacy Scale (EAS)

The propensity to advocate the importance of ethics in care was measured according to three Likert‐type items ranging from one (not applicable) to five (completely applicable) with the following response options: (a) “I think it's important—when there is a good reason to do so—to raise ethical aspects of care during patient care discussions,” (b) “I think it's important to be alert to the ethical implications of the medical treatment I provide,” and (c) “I think it's important for the organization where I work to focus explicit attention on the medical and ethical aspects of care.” A fourth question was added as well: “What is your opinion about applying ethical principles to medical care?” This question was measured with a semantic differential scale ranging from 0 (“completely useless”) to 100 (“very meaningful”). In order to combine the Likert‐type items with the semantic differential scale questions, the first three items were also converted along a continuum ranging from 0 to 100. Results of Principal Component Analysis with Varimax rotation demonstrated that the EAS construct was unidimensional, with factor loadings of 0.74, 0.79, 0.70, and 0.75, respectively. Results of reliability analysis indicated an acceptable level of internal consistency, as reflected by a Cronbach's alpha score of 0.72, with a mean inter‐item correlation coefficient (MIIC) of 0.40. Higher scores on the EAS reflect a higher propensity to advocate the importance of ethics in care.

#### 3.2.4 Behavioural control targeted at preventing harm (BCPH)

We measured behavioural control according to the following five items, which tapped the extent to which health practitioners were confident in their skills and alertness to prevent harm to the patient: (a) “I always feel responsible for proper patient care, even if the resources are insufficient,” (b) “My skill in assessing the needs of the patient always helps me in my work,” (c) “I can always properly assess whether and when a patient should be told the truth,” (d) “I can easily sense when a patient is not receiving proper care,” and (e) “In patient care, I am always aware of the balance between performing the task well and the risk of harm to the patient.” These items were answered along a 7‐point Likert scale ranging from 1 (completely disagree) to 7 (completely agree). Principal Component Analysis with Varimax rotation demonstrated that the BCPH scale was unidimensional, with factor loadings ranging from 0.54 to 0.83. The Cronbach's alpha score for the scale was 0.72, with a MIIC value of 0.37. Higher scores reflected greater perceived behavioural control targeted at preventing harm.

### 3.3 Statistical analysis

#### 3.3.1 Bivariate analysis

For categorical variables, we used the chi‐squared test (Fisher's exact tests for 2 × 2 contingency tables) and the difference between proportions test.^24^ For continuous variables, we used the Student's *t* test for independent samples. For correlation analysis, we used the parametric version of Pearson's *r*, as all of the continuous variables had been transformed toward normality.^25^

#### 3.3.2 Multivariate analysis

A regression‐based moderation analysis was applied. We computed the a priori minimum sample size (given an alpha value of 0.05, a power of 0.80, and an effect size of f2 = 0.15) to determine the appropriateness of conducting a moderation analysis. Based on the outcome (minimum = 68) and the sample size of the current study (n = 155), moderation analysis was deemed permissible. The moderation analysis was performed based on a built‐in bootstrap procedure of 5000 replications. All analyses, both bivariate and multivariate, were performed using IBM SPSS v. 25, and the regression‐based moderation analysis was conducted by using the PROCESS SPSS macro, version 3.4. The computation of the minimum required sample‐size for moderation analysis was performed using G*Power.^26^ To plot the cross‐over interaction effects of the unstandardized variables, we used an Excel spreadsheet made available by Professor James Gaskin.^27^

### 3.4 Ethical considerations

According to the statement by the Dutch Central Committee on Research Involving Human Subjects (www.ccmo.nl), no institutional review board approval was warranted for this type of survey with voluntary participation of professionals. An information letter sent to all respondents notified them of (a) the purpose of the study and (b) the voluntary nature of participation, and their right to stop participating in the study at any time. The respondents were also informed that their answers would be completely anonymous and that they would not be used for any purpose other than the study. Furthermore, the letter clearly addressed the expected average time needed to complete the questionnaires (45 minutes). This study was performed in accordance with the tenets of the Declaration of Helsinki.^28^ Only the first author (LK) had access to the encrypted data. The “Strengthening the Reporting of Observational Studies in Epidemiology” (STROBE) checklist was followed as a guideline for reporting on observational research.

## 4 RESULTS

### 4.1 Sociodemographic characteristics

An overview of the sociodemographic characteristics of the respondents is presented in Table 1. The average age of the respondents was 45.2 (± 9.1). The majority (70.3%) of the recruited sample were women. Less than half (46.5%) of the 155 respondents reported being religious, and 13.5% indicated a tendency to vote for a conservative political party. The results nevertheless did not reveal any statistically significant association (*χ* ^*2*^ = 3991, *df* = 1, *P* = 0.06) between religiosity and political preference. With respect to working environment, most (72.9%) of the respondents were employed in hospitals, with a smaller share (14%) working in family medicine (general practice) and the rest working either in mental healthcare (5.8%), care for people with mental disabilities (1.3%), or elsewhere (12.9%).

**TABLE 1:** Socio‐demographic characteristics of participants stratified to PAs and NPs

Sociodemographic characteristics		Physician assistant *N* = 88	Nurse practitioner *N* = 67	Total *N* = 155	(*P*‐value)
Age mean (SD)		42.5 (8.4)	48,8 (8.7)	45.2 (9.1)	< .001^a^
*Gender*	Female N (%)	56 (63.6)	53 (79.1)	109 (70.3%)	.05^b^
	Male N (%)	32 (36.4)	14 (20.9)	46 (29.7%)	
*Religion*	Not religious	48 (54.5)	35 (52.3)	83 (53.5%)	.54^c^
	No denomination but spiritual	3 (3.4)	4 (4.5)	7 (4.5%)	
	Christian	35 (39.8)	25 (37.3)	60 (38.7%)	
	Muslim	1 (1.1)	0	1 (0.7%)	
	Other religions	1	3 (4.5)	4 (2.6%)	
*Working environment*	Hospital N (%)	64 (72.7%)	49 (73.1%)	113 (72.9%)	.58^c^
	General practice N (%)	13 (14.8%)	7 (10.5%)	20 (12.9%)	
	Mental health care N (%)	3 (3.4%)	6 (9%)	9 (5.8%)	
	Disability care N (%)	1 (1.1%)	1 (1.5%)	2 (1.3%)	
	Other N (%)*)*	7 (8%)	4 (5.9%)	11 (7.1%)	
*Political orientation*	Conservative N (%)	15 (17%)	6 (9%)	21 (13.5%)	.14^c^
	Liberal N (%)	73 (83%)	61 (91%)	134 (86.5%)	

a Independent Sample's T‐test.

b Chi square test

c Difference between proportions test.

An overview of our main and sociodemographic variables is presented in Table 2, along with the correlations between them.

**TABLE 2:** Average scores and correlations across the scales themselves and with sociodemographic parameters

Sociodemographic parameters		(1)	(2)	(3)	(4)	(5)	(6)	(7)	
Age (1)									
Gender (2)		.041							
Working environment (3)		.023	−.012						
Religion (4)		.003	−.039	−.014					
Political orientation (5)		.167^*^	−.032	.151	−.160^*^				
Instruments	M (SD)								MIIC
Ethics Advocacy Scale (EAS) (6)	81.63 (12.1)	**.196** ^*^	−.071	.086	−.045	.081			.37
Behavioural Control targeted at Preventing Harm (BCPH) (7)	77.40 (10.15)	.039	.125	−.143	−.006	−.044	**.388** ^**^		.40
Reporting Reprehensible Conduct (RRC) (8) Vignette 1 Vignette 2	62.3 (33.5) 51.6 (24.8)	.012	.087	.013	−.007	.024	**.174** ^*^	**.190** ^*^	.34

Abbreviation: MIIC = mean inter‐item correlation coefficient.

* = Correlation is significant at the 0.05 level (2‐tailed); ** = Correlation is significant at the 0.01 level (2‐tailed).

### 4.2 Moderation analysis

**FIGURE 2:**
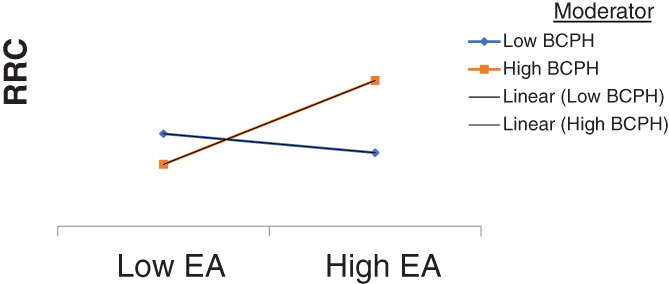
Plotting of the interaction effects (unstandardized) of BCPH on EA > RRC

To assess whether behavioural control targeted at preventing harm (BCPH, variable *M*) interacts with the relationship between *X* and *Y*, a regression‐based moderation analysis was performed. The overall model (see Figure 1) was significant: *R* ^*2*^ = .082, *F*(3, 151) = 4.49, *P* = .0047. The model that was tested did not reveal any main effects, either for EA (*B* = 481.6, *P* = .1586) or for BCPH (*B* = 309.7, *P =* .3886). It did reveal a significant interaction between EA and BCPH (*B* = 762.00, *t* [151] = 2.37, *P* = .012). As hypothesized, this interaction indicates that EA has a stronger positive effect on the likelihood of RRC when BCPH is high rather than low (See Figure 2). More precisely, EA has a statistically significant effect on reporting (Effect = .9892, *P* = .0091) only at the higher end of the scale (see Table 3, which displays the Johnson‐Neyman significance regions). These results suggest that EA does not increase the likelihood of RRC except when behavioural control is high, and that it has no effect at average or low levels. Given the significant correlation between “Age” and EA (see Table 2), we also tested the overall model by including age as a covariate. This had no impact on the effects.

**TABLE 3:** Conditional effect of EAS on BP‐RRC at values of the moderator BCPH (defined by Johnson‐Neyman significance region(s)

BCPH (raw scale scores)	BCPH (two‐step transformation scores)	Effect	se	t	p	LLCI	ULCI
	−2.4864	−1413.9	866.5	−1.63	.10	−3125.9	298
	−2.2378	−1224,4	793.5	−1.54	.12	−2792.2	343.4
	−1.9891	−1034.8	722	−1.43	.15	−2461.4	391.7
	−1.7405	−845.3	652.5	−1.29	.19	−2134.5	443.9
	−1.4919	−655.7	585.7	−1.12	.26	−1812.9	501.5
	−1.2432	−466.2	522.6	−.89	.37	−1498.7	566.4
	−.9946	−276.6	464.7	−.59	.55	−1194.8	641.6
	−.7459	−87.1	414.3	−.21	.83	−905.7	731.6
	−.4973	102.5	374.4	.27	.78	−637.2	842.2
	−.2486	292	348.5	.83	.40	−396.6	980.6
	.0000	481.6	339.9	1.41	.16	−190.1	1153.2
	.2486	671.1	349.9	1.91	.06	−20.3	1362.5
**≥ .80**	**.2824**	**696.8**	**352.7**	**1.97**	**.05**	**.0**	**1393.7**
	**.4973**	**860.7**	**377.1**	**2.28**	**.02**	**115.7**	**1605.7**
**≥ .83.3**	**.7459**	**1050.2**	**417.9**	**2.51**	**.01**	**224.4**	**1876**
**≥ 86.67**	**.9946**	**1239.8**	**469**	**2.64**	**<.01**	**313**	**2166.5**
	**1.232**	**1429.3**	**527.4**	**2.71**	**<.01**	**387.2**	**2471.3**
	**1.4919**	**1618.8**	**590.8**	**2.74**	**<.01**	**451.5**	**2786.2**
**≥ 93.33**	**1.7405**	**1808.4**	**657.9**	**2.75**	**<.01**	**508.6**	**3108.3**
**≥ 96.67**	**1.9891**	**1997.9**	**727.5**	**2.74**	**<.01**	**560.5**	**3435.5**
	**2.2378**	**2187.5**	**799.2**	**2.73**	**<.01**	**608.5**	**3766.6**
**100**	**2.4864**	**2377.1**	**872.3**	**2.72**	**<.01**	**653.7**	**4100.5**

*Note*: Bold are statistically significant regions at *P* < 0.05.

## 5 DISCUSSION

The objective of our study was to assess whether the reporting of ethical mistakes committed by colleagues could be predicted by the extent to which healthcare professionals regard ethical care as important and the extent to which they perceive to have behavioural control. More precisely, we hypothesized that converting motivation to report reprehensible conduct requires that the individual must feel capable of doing so. We therefore expected behavioural control targeted at preventing harm (BCPH) to moderate the effect of ethics advocacy (EA) on reporting behaviour. The results of our study provide evidence to confirm this hypothesis.

According to our results, although EA was correlated with “reporting reprehensible conduct in care” (RRC), it had no statistically significant main effect on RRC in the overall regression‐based moderation model. The hypothesis that BCPH acts as a “facilitator” to strengthen the relationship between EA and RRC was confirmed. The interaction between EA and BCPH showed that the positive effect of EA on RRC was only present for people with an above‐average perception of control (BCPH score ≥ 80, representing the 33.6% highest BCPH scorers). For people with an average or below average perception of control, EA did not increase the intention to report. These results suggest that the motivation to act morally based on EA is not sufficient to ensure actual reporting behaviour. The professional must also be convinced that reporting reprehensible conduct will be of benefit to those who have been negatively affected. In other words, a sufficient level of *behavioural control* is needed in order to ensure that a professional will feel able to convert the motivation to report into the actual reporting behaviour. These results are in line with Bandura's claim that perceived behavioural control helps individuals to determine what to do with the knowledge and skills they possess.^14^ Our data suggest that, within the context of healthcare, the perception of having control over doing no harm to the patient can help health professionals to act upon the importance that they attach to moral values in care by reporting any reprehensible conduct of colleagues that they might observe. In this regard, BCPH facilitates the translation of the motivation to report morally questionable behaviour of colleagues into action.

Our findings that both ethics advocacy and behavioural control play an important role in the likelihood of reporting reprehensible conduct can also be understood within the context of the Theory of Planned Behaviour.^17^ In their systematic review, Godin and Kok (1996) describe 56 studies reporting that “planned behaviour” has a statistically significant correlation with both “attitude” (*r* = .22 to .77) and “perceived behavioral control” (*r* = .14 to .85). These correlations were found among a wide variety of study subjects and domains, including (a) addiction (eg, quitting smoking), (b) exercising behaviour (eg, initiating sport activities for health benefits), (c) oral hygiene behaviour (eg, preventing dental decay by brushing frequently) and (d) health‐risk prevention behaviour (eg, condom use to prevent HIV).

The outcomes of the present study contribute to the literature on whistleblowing. We developed and tested a context‐specific measure of whistleblowing explicitly for individual healthcare providers (eg, PAs and NPs). These efforts were prompted largely by a recently published narrative review by Blenkinsopp and colleagues, which identifies 58 studies addressing the phenomenon of whistleblowing in healthcare at least to some extent,^29^ with the greatest share of these studies focusing exclusively on nursing populations. This is problematic, as the findings for nurses may not generalize to other health professions, given that nurses usually work in teams, in addition to having their own professional culture, interactions, norms, and values. Moreover, their relatively small range of decision authority may hamper whistleblowing behaviour. The current study investigates whistleblowing behaviour among PAs and NPs, whose autonomous, full‐practice authority should logically make them more likely to engage in whistleblowing.^30^ Our findings show that, even in light of such professional authority, these practitioners still require a higher‐than‐average level of perceived behavioural control in order to translate their motivation to act morally into actual behaviour.

### 5.1 Strengths and limitations

One strength of this study is that it is based on a representative sample in terms of gender and age that reflects the demographics of both the NP and PA workforces in the Netherlands.^31^ For this reason, the results can be generalized to a certain degree. The findings obtained among these autonomous PAs and NPs could conceivably also be applied to professionals with comparable independent treatment relationships (eg, medical doctors, physical therapists, speech therapists, or dental hygienists).

In methodological terms, another strength of our study is the sample size—155 respondents—which is well above the minimum required for moderation analysis (*n* = 68).^26^ In addition, despite the cross‐sectional nature of the data, the Harman's single‐factor analysis indicated that a single factor accounted for only 28.7% of the total variance. Given the maximum threshold of 50%, common‐method variance thus had little or no effect on the conclusions.^32^

Our study is also subject to several limitations. First, the cross‐sectional nature of the data did not allow us to assess the stability (ie, test‐retest) of the instruments. Second, even though the correlations between RRC, EA, and BCPH were statistically significant, their explained variances were relatively low. It should therefore be clear that many other factors*—which were not included in this study*—could explain or influence whistleblowing behaviour. Further exploration is therefore needed. Another possible limitation has to do with the low reliability of the two vignettes in the RCC measure (Cronbach's alpha value of 0.51). As previously described, however, the mean inter‐item correlation (MIIC) of 0.34 fell well within the specified range (≥.25 to ≤.55), thereby indicating an acceptable level of homogeneity for the two vignettes.^33^ Nevertheless, the inclusion of more vignettes could offer a solution for achieving a high Cronbach's alpha value.^34^, ^35^ According to the formula proposed by Nunally^36^ (page 225) for estimating the number of items (*k*) necessary to obtain the required alpha value of 0.80, the current RRC scale should be extended with six vignettes that tap particular aspects of the underlying construct. This provides an avenue for continuing research on this specific indicator of whistleblowing within the context of healthcare.

## 6 IMPLICATIONS

The healthcare landscape is changing rapidly. More specifically, patients are becoming more vocal, measures are being taken to keep care affordable, and sociodemographic processes (including population aging) are exerting pressure on the balance between the demand for and supply of care. All of these factors are combining to increase the prevalence of situations in which moral considerations come into play. According to our results, behavioural control targeted at preventing harm (BCPH) plays a pivotal role in the ethical decision‐making process. More specifically, BCPH acts as a facilitator, strengthening the relationship between ethics advocacy and the likelihood of reporting reprehensible conduct in care. In other words, a high level of perceived behavioural control is needed in order to ensure that people will act according to their values. It is therefore essential to foster the sense of behavioural control among healthcare professionals. One way could be to increase their knowledge of or experience with morally delicate circumstances. Exposing students to ethical dilemmas from the early phases of their training (eg, through frequent fictitious patient encounters) could help their behavioural control to mature as their training progresses. The complexity of the ethical situations addressed during such educational sessions could conceivably be coordinated to correspond to where the students are in their training programs at that moment. As students become more comfortable in coping with ethical dilemmas, they are likely to grow more confident in their ability to prevent harm in care. This could help them to act on their moral values upon encountering reprehensible conduct in their future professional lives.

## CONFLICT OF INTEREST

The authors declare no conflict of interest.

## AUTHOR CONTRIBUTIONS

Luppo Kuilman: study conception/design; acquisition (and storage) of data; analysis and interpretation of data; writing and critical revision of the manuscript.

Gerard Jansen: study conception/design; drafting manuscript; critical revision.

Laetitia Mulder: study conception/design of theoretical model; drafting of the manuscript; critical revision.

Petrie Roodbol: study conception/design; critical revision.

## Supporting information

**Data S1.** Vignettes 1 and 2 on reporting reprehensible conduct in care.
