# Supplementary material for: Facilitating and motivating factors for reporting reprehensible conduct in care: A study among nurse practitioners and physician assistants in the Netherlands
Source: J Eval Clin Pract. 2020 Aug 20;27(4):776–84. doi: 10.1111/jep.13462 (PMC8359255; doi:10.1111/jep.13462)
Supplement: Supplementary file 1 — Data S1. Vignettes 1 and 2 on reporting reprehensible conduct in care. [file JEP-27-776-s001.pdf]

Appendix 1: Vignettes 1 and 2 on Reporting Reprehensible Conduct in Care

Vignette 1. Nobody needs to know … heart for a heart.

Suppose that you are working as a physician assistant at a thoracic surgery ward where heart transplants are performed. Willemijn, a young patient, is very ill. If she does not receive a donor heart soon, she will die. You are very concerned about her. She is so gentle and cheerful, despite her predicament. She is the darling of the entire ward. Eurotransplant notifies the ward that a donor heart is available. According to the waiting list, Mr. Van der Sluis is eligible for it. Both he and Willemijn are a match. Mr. Van der Sluis has been rude to almost everyone on the ward staff. Your colleague Hans, who – unlike you – has access to the data, tells you in confidence that he will use the computer to change the rank order in favor of Willemijn. He is certain that this will remain unnoticed and asks you not to tell anybody.

On the line below, please indicate to what degree you are inclined to discuss this switch with a third party.

Not at all **Certainly**

|____|

Vignette 2. So much morphine, that isn't palliative sedation …

You are working as a physician assistant at the practice of a dispensing physician. One of the patients has metastasized lung cancer. You saw this patient recently because he complained of increasing pain in the right upper quadrant. You then also discussed the patient's views on euthanasia and palliative sedation. The patient said that he was opposed to euthanasia but did not want to die choking either. He will allow the GP to put him to sleep and then 'let go softly'. This morning, you receive word that the patient died overnight. When you look at his file, you notice the following entry: A96.01 'natural death'; in the pharmacy module, you read that three 1 mL (10 mg/mL) ampoules of morphine have been used. Digging further, you notice that eight 5 mL (20 mg/mL) ampoules – a total of 800 mg – of morphine were written off in the opiate ledger today because they 'fell on the floor and broke'. This is highly unusual. You strongly suspect that the GP has ignored the legislative obligations concerning euthanasia and palliative sedation. You confront the GP with your findings. The GP says that in this acute situation, there was no time to observe the palliative sedation protocol.

On the line below, please indicate to what extent you would be inclined to report this case to the Health Care Inspectorate.

Not at all **Certainly**

|____|
